# Risk Factors and Molecular Features of Sequence Type (ST) 131 Extended-spectrum β-Lactamase-producing *Escherichia coli* in Community-onset Bacteremia

**DOI:** 10.1038/s41598-017-14621-4

**Published:** 2017-11-07

**Authors:** Hyunsoo Kim, Young Ah Kim, Yoon Soo Park, Min Hyuk Choi, Gyeong In Lee, Kyungwon Lee

**Affiliations:** 1 Department of Laboratory Medicine, National Police Hospital, Seoul, 05715 Republic of Korea; 2 Department of Laboratory Medicine, National Health Insurance Service Ilsan Hospital, Goyang, 10444 Republic of Korea; 3 Department of Internal Medicine, National Health Insurance Service Ilsan Hospital, Goyang, 10444 Republic of Korea; 4 Department of Laboratory Medicine and Research Institute of Bacterial Resistance, Yonsei University College of Medicine, Seoul, 03722 Republic of Korea

## Abstract

Due to the spread of a single CTX-M-type extended-spectrum β-lactamase (ESBL) clone of sequence type (ST) 131, community-onset bacteremia caused by ESBL-producing *Escherichia coli* has increased dramatically. We evaluated the risk factors and molecular features of ESBL-producing *E. coli* ST131 clones isolated from Korean patients with community-onset bacteremia. We collected a total of 124 ESBL-producing *E. coli* isolates from blood in patients with community-onset bacteremia over a 2 year-period. Among 124 patients, the number of community-associated bacteremia cases was 57 (46%). ST131 strains accounted for 49.1% (28/57) of community-associated bacteremia cases and 44.8% (30/67) of healthcare-associated community-onset bacteremia cases. Among 58 ST131 strains, nine isolates were shown to harbor O16-H41, and 61.1% (30/49) of O25 had *H30Rx*. In a multivariate analysis, independent risk factors for acquisition of ST131 isolates over non-ST131 isolates were underlying diabetes mellitus and absence of prior chemotherapy history. The most common ESBL genotype was CTX-M-15 (46.0%), followed by CTX-M-14 (37.1%). A considerable proportion of community-onset ESBL-producing *E. coli* bacteremia was observed. ST131 clones appear to be associated with the spread of community-associated bacteremia exhibiting high antimicrobial resistance and highly virulent *H30Rx* traits, which could become a major public health concern in Korea.

## Introduction

Extended-spectrum β-lactamase (ESBL)-producing *Escherichia coli* has become widespread in hospitals around the world since the late 1980s^1^, but the sudden worldwide increase in communities is mainly due to CTX-M-type ESBLs (especially CTX-M-15)-producing sequence type (ST) 131^2,3^. The most common type of infection by ESBL-producing *E. coli* is urinary tract infection with good clinical outcomes^4,5^. Life-threatening infections such as bacteremia have not drawn public attention until now, but the emergence of community-onset bacteremia by ESBL-producing *E. coli* ST131 clones in Korea has been a concern in recent reports^6–8^. ST131 ESBL-producing *E. coli* from bacteremia cases possessed more virulence traits and showed more multidrug resistance patterns than non-ST131 isolates^7^. Recent molecular epidemiology showed that *H30Rx* subsets within ST131-O25-*H30* subclones were associated specifically with fluoroquinolone resistance, and CTX-M-15 was widely detected in Korea (37% of total ST131 isolates)^8^. Risk factors of community-onset bacteremia by *E. coli* through a comparison of ESBL and non-ESBL groups have already been evaluated^9^, but the clinical impacts of ST131 have not been elucidated in community-onset bacteremia as far as we know. The potential spread of ESBL-producing *E. coli* causing blood stream infections is a challenge for the management of community-associated infections, so this study could be informative regarding current molecular epidemiologic shifts in community-onset bacteremia and could lead to better infection control strategies.

## Methods

### Design and Setting

We collected 124 non-duplicated (except initial isolate from each patient duplicate series) ESBL-producing *E. coli* blood culture isolates from consecutively encountered patients with community-onset bacteremia, as outpatients or within 48 hours of admission between 2013 and 2014 in Severance hospital, which is one one tertiary teaching hospital in Seoul, Korea. Community-associated bacteremia, risk factors, associated disease, and source of infection followed the definitions used in our previous study^9^. Briefly, healthcare-associated infections were classified in accordance to the definition by Friedman *et al*. with some modifications^10^. Any of the following criteria were considered as healthcare-associated infections: intravenous therapy, wound care, or nursing care received at home 30 days before bloodstream infection; attendance at a hospital or hemodialysis clinic, or receipt of intravenous chemotherapy 30 days before bloodstream infection; >48 hours of hospital admission or performance of invasive procedures such as urinary catheter, endoscopy, and naso-gastric tube 90 days before bloodstream infection; or residence at nursing home or long-term care facility. Thirty-day mortality was defined as death for any reason within 30 days after the onset of the bacteremia. Immunosuppression was defined as follows: therapy of prednisolone or an equivalent drug with a dosage of at least 10 mg/day for 15 days, and chemotherapy or radiotherapy within 6 months before the bacteremia^11^.

### Microbiological Analysis

Identification, ESBL screening, and susceptibility testing were performed using the automated analyzer Vitek 2 system (bioMérieux, Marcy l′E’toile, France), and results of susceptibility were interpreted using the CLSI^12^. For detection of ST131, all isolates were screened via PCR for O16-ST131 and O25-ST131^13^. *FimH* type and *H30Rx* were determined using PCR and sequencing^14,15^. ESBL genotypes were determined via PCR and sequencing^16^. Sequence types (ST) were confirmed with full multilocus sequence typing (MLST) for representative isolates of community-associated bacteremia group^17,18^. Pulsed-field gel electrophoresis (PFGE) was performed as described in our previous study^9^. The patterns were analyzed using InfoQuest FP software (Bio-Rad) to generate a dendrogram based on the unweighted pair group method, with an arithmetic average (UPGMA) from the Dice coefficient with 1% band position tolerance and 0.5% optimization settings. A PCR-based replicon typing (PBRT) was schemed by targeting the replicons of major plasmid families occurring in Enterobacteriaceae (HI2, HI1, I1-γ, X, L/M, N, FIA, FIB, FIC, W, Y, P, A/C, T, K, B/O) for representative isolates^19^.

### Statistical Analysis

Continuous variable, such as age, was analyzed by using the Mann-Whitney U test. The chi-squared test was used for the comparative analysis of categorical variables in order to determine independent risk factors. Odds ratio (OR) and 95% confidence interval (CI) values were calculated for binomial variables. Variables for which the *P* values were less than 0.1 in univariate analyses were included in a multivariate logistic regression analysis model to determine independent risk factors for acquisition of ST131 isolates. Statistical significance was defined as *P* < 0.05. SPSS 17.0 software (SPSS, Chicago, IL, USA) was used for univariate analyses and multivariate analyses.

### Data Availability

All data generated or analysed during this study are included in this published article.

## Results

### Clinical features of patients with community-onset ESBL-producing *E. coli* bacteremia

Of 124 total patients with ESBL-producing *E. coli* community-onset bacteremia, 57 (46%) had community-associated bacteremia and the others had healthcare-associated bacteremia. There were fewer patients with a Charlson comorbidity index score of 2 or above in the community-associated bacteremia group than in the healthcare-associated bacteremia group. The mortality rate in patients with community-associated bacteremia (5.3%) was lower than that in patients with healthcare-associated bacteremia (22.8%, Table 1). However, no statistical difference was found between mortality rates of ST131 and non-ST131 (Table 4). Also, there was no statistical difference between mortality rates of *H30Rx* subclone and non-ST131 (*P* = 0.090).

**Table 1:** Clinical features of patients with community onset ESBL-producing *E. coli* bacteremia: Univariate Analysis.

Clinical features	CA (n = 57)	HA (n = 67)	OR (95% CI)	*P* value
Age in years, median (IQR)	72.0 (61.0–77.0)	68.0 (56.5–75.0)		0.127
Male sex	20 (35)	32 (48)	0.597 (0.289–1.232)	0.163
Associated disease				
Diabetes mellitus	20 (35)	22 (33)	1.105 (0.525–2.330)	0.792
Heart failure	3 (5)	1 (1)	2.846 (0.326–24.891)	0.344
Chronic pulmonary disease	1 (2)	1 (1)	1.176 (0.072–19.239)	0.909
Chronic renal insufficiency	6 (11)	12 (18)	0.561 (0.197–1.595)	0.278
Liver cirrhosis	1 (2)	2 (3)	0.696 (0.065–7.403)	0.764
Chemotherapy	8 (14)	22 (33)	0.347 (0.141–0.853)	0.021
Vascular disease	8 (14)	9 (13)	1.057 (0.379–2.948)	0.915
Transplantation	3 (5)	3 (4)	1.183 (0.229–6.105)	0.841
Immunosuppression	3 (5)	3 (4)	1.183 (0.229–6.105)	0.841
Major surgery 30 d before infection	16 (28)	20 (30)	0.921 (0.423–2.008)	0.837
Charlson comorbidity index ≥ 2	24 (42)	48 (72)	0.294 (0.139–0.620)	0.001
Device				
Urinary catheter	0 (0)	10 (15)	0.048 (0.002–0.955)	0.047
Tracheostomy/intubation	0 (0)	2 (3)	0.228 (0.005–9.549)	0.438
Nasogastric tube	0 (0)	3 (4)	0.16 (0.005–4.998)	0.297
Any device	0 (0)	11 (16)	0.043 (0.002–0.841)	0.038
Previous antibiotics within last month				
Penicillin	1 (2)	6 (9)	0.251 (0.036–1.734)	0.161
Cephalosporin				
First generation	0 (0)	2 (3)	0.228 (0.005–9.549)	0.438
Second generation	1 (2)	4 (6)	0.375 (0.048–2.929)	0.350
Third generation	5 (9)	10 (15)	0.574 (0.185–1.776)	0.335
Carbapenem	1 (2)	8 (12)	0.186 (0.029–1.201)	0.077
Fluoroquinolone	1 (2)	9 (13)	0.164 (0.026–1.033)	0.054
Any antibiotics	9 (16)	35 (52)	0.179 (0.077–0.420)	<0.001
Source of infection				
Primary	11 (19)	21 (31)	0.535 (0.232–1.232)	0.142
Urinary	39 (68)	37 (55)	1.737 (0.831–3.628)	0.142
Hepatobiliary	5 (9)	5 (7)	1.191 (0.327–4.339)	0.791
Gastrointestinal	2 (4)	0 (0)	6.074 (0.145–254.296)	0.344
Respiratory	0 (0)	5 (7)	0.099 (0.004–2.404)	0.155
Polymicrobial infection	4 (7)	8 (12)	0.589 (0.169–2.046)	0.405
ST131 clone	28 (49)	30 (45)	1.188 (0.585–2.411)	0.634
Septic shock/severe sepsis	11 (19)	21 (31)	0.535 (0.232–1.232)	0.142
Pitt bacteremia score ≥ 2	16 (28)	18 (27)	1.064 (0.482–2.346)	0.878
30-day mortality	3 (5)	13 (19)	0.259 (0.073–0.923)	0.037

Data are no. (%) of patients. CA, community-associated; HA, healthcare-associated; OR, odds ratio; CI, confidence interval.

### Microbiological Analysis

**Figure 1:**
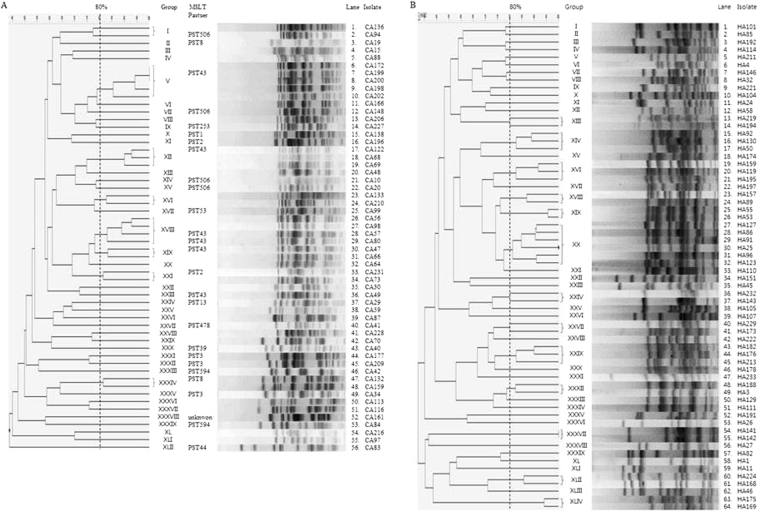
Pulsed-field gel electrophoresis (PFGE) of *XbaI*-restricted DNA of 120 community-onset ESBL-producing *E. coli*: (**A**) 56 community-associated ESBL-producing *E. coli*; (**B**) 64 healthcare-associated ESBL-producing *E. coli*.

The antimicrobial susceptibilities of community-onset ESBL–producing *E. coli* were similar between the community-associated and healthcare-associated bacteremia groups, except for the results of ceftazidime and aztreonam, which were associated with more resistance in the healthcare-associated bacteremia group (Table 2). Globally, epidemic ST131 strains accounted for 49.1% (28/57) of community-associated bacteremia cases and 44.8% (30/67) of healthcare-associated bacteremia cases. Of 58 total ST131 strains, nine O16-H41 strains were detected, and 61.1% (30/49) of O25-*H30* strains had *H30Rx* (Table 3). The most common ESBL genotype was CTX-M-15 (46.0%, 57/124), followed by CTX-M-14 (37.1%, 46/124). PFGE patterns did not show a dominant clonality in community-associated or community-onset healthcare-associated bacteremia (Fig. 1). Eighteen “Pasteur” sequence type (PST) isolates other than ST131 from community-associated bacteremia (based on representative PFGE patterns) were PST3 (n = 3), PST8 (n = 3), PST2 (n = 2), PST594 (n = 2), and one each for PST13, PST39, PST44, PST53, PST253, PST478, PST666, and PST unknown by the Pasteur MLST scheme. A PBRT was conducted for 16 representative isolates of community-onset ESBL–producing *E. coli*. Among these 16 isolates, only nine succeeded in conjugation. Of the nine isolates, five (two of CA group and three of HA group) were positive for IncFIA replicon, two showed positive for IncI1-Iγ, and the rest were negative for all tested replicons. The two types of replicons almost equally belonged to CA group (n = 3) and HA group (n = 4).

**Table 2:** Comparison of antimicrobial resistance between CA and HA in the community-onset ESBL–producing *E. coli*: Univariate Analysis.

Antimicrobial agent	CA (n = 57)	HA (n = 67)	OR (95% CI)	*P* value
Ampicillin-sulbactam	46 (81)	54 (81)	1.002 (0.410–2.449)	0.997
Piperacillin-tazobactam	0 (0)	3 (4)	0.160 (0.005–4.998)	0.297
Cefotaxime	55 (96)	67 (100)	0.165 (0.004–6.892)	0.344
Ceftazidime	38 (67)	57 (85)	0.361 (0.152–0.857)	0.021
Cefepime	35 (61)	49 (73)	0.590 (0.276–1.260)	0.173
Meropenem	0 (0)	0 (0)	—	—
Ertapenem	0 (0)	0 (0)	—	—
Levofloxacin	42 (74)	53 (79)	0.743 (0.323–1.710)	0.486
Aztreonam	43 (75)	60 (90)	0.372 (0.139–0.994)	0.049
Amikacin	0 (0)	0 (0)	—	—
Gentamycin	24 (42)	34 (51)	0.710 (0.349–1.446)	0.346
Trimethoprim-sulfamethoxazole	30 (53)	37 (55)	0.902 (0.444–1.832)	0.776

Data are no. (%) of resistant isolates. CA, community-associated; HA, healthcare-associated; OR, odds ratio; CI, confidence interval.

**Table 3:** Genetic subgroup and result of susceptibility test to fluoroquinolone of the community-onset ESBL–producing *E. coli*.

	CA (n = 57)		HA (n = 67)	
	O25b-ST131 (n = 22)	O16-ST131 (n = 6)	O25b-ST131 (n = 27)	O16-ST131 (n = 3)
FimH type				
Null	0	1	0	0
H30	22	0	27	0
H41	0	5	0	3
CTX-M type				
CTX-M-14	6	3	5	2
CTX-M-15	15	2	15	1
CTX-M-27	1	1	6	0
Other CTX-M	0	0	1	0
Susceptibility test to fluoroquinolone				
Resistance	22	2	27	2
Susceptibility	0	4	0	1

Data are no. of resistant isolates. CA, community-associated; HA, healthcare-associated.

### Risk factors of acquisition of ST131 in community-onset ESBL-producing *E. coli* bacteremia

According to a univariate analysis, independent risk factors for acquisition of ST131 isolates over non-ST131 isolates were diabetes mellitus (OR, 2.495; 95% CI, 1.162–5.356; *P* = 0.019), chronic renal insufficiency (OR, 3.317; 95% CI, 1.116–9.856; *P* = 0.031), absence of active cancer (OR, 2.740; 95% CI, 1.314–5.682; *P* = 0.007), absence of history of prior chemotherapy (OR, 3.003; 95% CI, 1.222–7.353; *P* = 0.016), and absence of history of surgery (OR, 3.003; 95% CI, 1.222–7.353; *P* = 0.016) (Table 4). A multivariate analysis showed that diabetes mellitus (OR, 2.347; 95% CI, 1.074–5.128; *P* = 0.032) and absence of prior chemotherapy history (OR, 2.882; 95% CI, 1.147–7.246; *P* = 0.024) were significant risk factors for acquisition of ST131 isolates. Antimicrobial susceptibility of the community-onset ESBL–producing *E. coli* appeared similar between the ST131 and non-ST131 bacteremia groups, except for the results of levofloxacin, which were associated with more resistance in the ST131 bacteremia group (Table 5).

**Table 4:** Risk factors of acquisition of ST131 in community-onset ESBL-producing *E. coli* bacteremia: Univariate Analysis.

Risk factors	ST131 (n = 58)	Non-ST131 (n = 66)	OR (95% CI)	*P* value
Age in years, median (IQR)	73.5 (61.5–80.0)	68.5 (59.0–74.0)		0.057
Male sex	24 (41)	28 (42)	0.959 (0.469–1.961)	0.909
Associated disease				
Diabetes mellitus	26 (45)	16 (24)	2.495 (1.162–5.356)	0.019
Heart failure	0 (0)	4 (6)	0.119 (0.004–3.174)	0.204
Chronic pulmonary disease	1 (2)	1 (2)	1.139 (0.070–18.621)	0.928
Chronic renal insufficiency	13 (22)	5 (8)	3.317 (1.116–9.856)	0.031
Liver cirrhosis	1 (2)	2 (3)	0.673 (0.063–7.163)	0.743
Chemotherapy	8 (14)	22 (33)	0.333 (0.136–0.818)	0.016
Vascular disease	10 (17)	7 (11)	1.718 (0.609–4.844)	0.306
Transplantation	1 (2)	5 (8)	0.292 (0.040–2.119)	0.223
Immunosuppression	3 (5)	3 (5)	1.145 (0.222–5.906)	0.872
Major surgery 30 d before infection	11 (19)	25 (38)	0.394 (0.173–0.895)	0.026
Charlson comorbidity index ≥ 2	30 (52)	42 (64)	0.617 (0.301–1.266)	0.188
Device				
Urinary catheter	5 (9)	5 (8)	1.150 (0.316–4.189)	0.833
Tracheostomy/intubation	1 (2)	1 (2)	1.139 (0.070–18.621)	0.928
Nasogastric tube	0 (0)	3 (5)	0.155 (0.005–4.836)	0.288
Any device	5 (9)	6 (9)	0.957 (0.276–3.315)	0.945
Previous antibiotics within last month				
Penicillin	4 (7)	3 (5)	1.498 (0.322–6.965)	0.606
Cephalosporin				
First generation	1 (2)	1 (2)	1.139 (0.070–18.621)	0.928
Second generation	1 (2)	4 (6)	0.363 (0.046–2.832)	0.335
Third generation	8 (14)	7 (11)	1.335 (0.453–3.940)	0.600
Carbapenem	3 (5)	6 (9)	0.587 (0.142–2.422)	0.461
Fluoroquinolone	2 (3)	8 (12)	0.305 (0.066–1.398)	0.126
Any antibiotics	18 (31)	26 (39)	0.698 (0.332–1.468)	0.344
Source of infection				
Primary	12 (21)	20 (30)	0.610 (0.268–1.389)	0.239
Urinary	40 (69)	36 (55)	1.829 (0.875–3.823)	0.108
Hepatobiliary	6 (10)	4 (6)	1.720 (0.463–6.398)	0.418
Gastrointestinal	0 (0)	2 (3)	0.221 (0.005–9.242)	0.428
Respiratory	0 (0)	4 (6)	0.119 (0.004–3.174)	0.204
Polymicrobial infection	5 (9)	7 (11)	0.815 (0.245–2.718)	0.740
CA	28 (48)	29 (44)	1.188 (0.585–2.411)	0.634
Septic shock/severe sepsis	16 (28)	16 (24)	1.188 (0.531–2.658)	0.675
Pitt bacteremia score ≥ 2	12 (21)	22 (33)	0.532 (0.236–1.200)	0.128
30-day mortality	4 (7)	12 (18)	0.360 (0.111–1.163)	0.088

Data are no. (%) of patients. CA, community-associated; OR, odds ratio; CI, confidence interval.

**Table 5:** Comparison of antimicrobial resistance between ST131 and non-ST131 in the community-onset ESBL–producing *E. coli*: Univariate Analysis.

Antimicrobial agent	ST131 (n = 58)	Non-ST131 (n = 66)	OR (95% CI)	*P* value
Ampicillin-sulbactam	50 (86)	50 (76)	1.941 (0.765–4.925)	0.163
Piperacillin-tazobactam	2 (3)	1 (2)	1.933 (0.182–20.565)	0.585
Cefotaxime	58 (100)	64 (97)	4.531 (0.108–189.767)	0.428
Ceftazidime	46 (79)	49 (74)	1.315 (0.567–3.048)	0.523
Cefepime	41 (71)	43 (65)	1.281 (0.600–2.735)	0.522
Meropenem	0 (0)	0 (0)	—	—
Ertapenem	0 (0)	0 (0)	—	—
Levofloxacin	53 (91)	42 (64)	5.608 (2.014–15.616)	0.001
Aztreonam	49 (84)	54 (82)	1.195 (0.464–3.077)	0.712
Amikacin	0 (0)	0 (0)	—	—
Gentamycin	29 (50)	29 (44)	1.271 (0.626–2.580)	0.507
Trimethoprim-sulfamethoxazole	28 (48)	39 (59)	0.651 (0.319–1.325)	0.236

Data are no. (%) of resistant isolates. OR, odds ratio; CI, confidence interval.

### Risk factors of acquisition of *H30Rx* subclone in community-onset ESBL-producing *E. coli* bacteremia

Based on univariate analysis, independent risk factors for acquisition of *H30Rx* subclone over non-ST131 isolates were diabetes mellitus (OR, 4.33; 95% CI, 1.77–10.99; *P* = 0.002), chronic renal insufficiency (OR, 4.99; 95% CI, 1.55–17.80; *P* = 0.009), absence of active cancer (OR, 3.33; 95% CI, 1.37–8.33; *P* = 0.010), and absence of surgical history (OR, 4.17; 95% CI, 1.41–14.29; *P* = 0.017). Multivariate analysis indicated no significant risk factors for acquisition of *H30Rx* subclone.

## Discussion

We have already reported that independent risk factors of community-onset ESBL-producing *E. coli* bacteremia are healthcare-associated infection, malignancy, urinary tract infection, hepatobiliary tract infection, third generation cephalosporin usage during the preceding three months, and severe sepsis/septic shock^9^. The most common types of ESBL causing community-onset bacteremia were CTX-M-15 and CTX-M-14, and the most commonly defined sequence type (ST) was ST131 (11/60, 18.3%) during the study period (from 2005 to 2009)^9^.

In this study, recent epidemiology (observed between 2013 and 2014) changed with a remarkable increase of ST131 in community-onset bacteremia (from 18.3 to 46.8%). A recent dramatic increase of ST131 has been reported worldwide, causing serious concern^20,21^. This epidemiologic shift explains the recent increase of community-onset bacteremia in that the ST131-O25-*H30* subclone is associated with persistent infections and later adverse outcomes, which are independent of multidrug resistance and the association with compromised hosts^20^. Although this earlier study differed in its study population compared to our study (community-onset urinary tract infection in the majority vs. community-onset bacteremia only), it supports the hypothesis that *H30* has distinctive properties that allow it to evade host defenses and cause delayed complications^20^.

In this study, the *H30Rx* subclone was prevalent as ST131-O25-*H30* (61.1%, 30/49), and O16-*H41* strains were not negligible (15.5% of the total ST131 strains). Serotype O16 was assigned to ST131 by the Achtman MLST scheme, but was distinct from the classic ST131-O25 that has resistance to ampicillin, gentamicin, and trimethoprim-sulfamethoxazole, and showed susceptibility to fluoroquinolones and extended-spectrum cephalosporin^13^. This study suggests that ST131 isolates show more multidrug resistance patterns than non-ST131 isolates. A recent multicenter surveillance study of Korea reported the similar result, although the resistance rate to piperacillin-tazobactam was much higher than that seen in our results^7^. The single, rapidly expanding ST131 subclone *H30-Rx*, which is strongly associated with fluoroquinolone resistance and CTX-M-15 ESBL, is the most resistant ST131 strain^14^. The spread of the O16 and *H30Rx* clones in the Korean community could be important for transmission prevention-based control strategies because of their resistance to effective antibiotics.

PFGE patterns did not show a dominant clone for community-associated or healthcare-associated bacteremia, and PSTs were also varied by the Pasteur MLST scheme. IncFIA and IncI1-Iγ replicons were distributed evenly in community-associated and healthcare-associated bacteremia. This is suggestive of multiple evolutionary processes in the extraintestinal *E. coli* community in the course of the emergence of dominant ST131 clones.

Many studies on risk factors for ST131 have been reported worldwide since 2013^22–25^. However, despite the high incidence of *E. coli* ST131 ESBLs in Korea, the characteristics have rarely been investigated. We investigated risk factors for acquisition of ST131 in patients with community-onset ESBL-producing *E. coli* causing bloodstream infections, and we adjusted confounding variables such as severity of underlying disease, and co-morbidities. Although diabetes mellitus and absence of prior chemotherapy history were significantly associated with acquisition of ST131 clones, in the present study, other underlying disease and co-morbidities were similar between the ST131 group and the non-ST131 group. These results suggest that *E. coli* ST131 strains producing ESBLs have disseminated in both the community and in hospitals in Korea. Previous studies investigated the risk factors for colonization or infection caused by isolates of ST131 *E. coli* ^22–26^. According to such studies, there were many risk factors for acquisition of ST131 *E. coli* such as recent surgery, unknown source of bacteremia, old age, long-term residency at care facility, urinary tract infection within the previous 30 days, complex infection, previous receipt of extended-spectrum cephalosporins and macrolides or fluoroquinolones, female gender, diabetes mellitus, bedridden status, secondary bacteremia, and nonuse of urinary catheter. Risk factors were different in each study, including the current study. Our interpretation is that the markedly different population in each study caused the difference in risk factors for acquisition of ST131 *E. coli*. There are some limitations in this study in that it was conducted at a single university hospital; therefore the results cannot be generalized to community hospitals and other university hospitals with different settings. Although community-onset healthcare-associated bacteremia group included patients with records of previous hospitalization (within the last 3–6 months) and residency at long-term care facilities, we did not analyze them separately.

In conclusion, a considerable proportion of community-onset ESBL-producing *E. coli* bacteremia was observed. ST131 clones appear to be associated with the spread of community-associated bacteremia exhibiting high antimicrobial resistance and highly virulent *H30Rx* traits, which could become a major public health concern in Korea. The potential spread of ESBL-producing *E. coli* causing blood stream infections is a challenge for the management of community-associated infections, so this study could be informative regarding current molecular epidemiologic shifts in community-onset bacteremia and could lead to better infection control strategies.
